# Quantifying the Acute Toxicity of Alpha-Synuclein Membrane-Accumulation in a Cell-Based Assay

**DOI:** 10.21203/rs.3.rs-8576084/v1

**Published:** 2026-01-21

**Authors:** Baoyi Li, Amirah K. Anderson, Oren A. Levy, Nagendran Ramalingam, Ulf Dettmer

**Affiliations:** Wycombe Abbey; Brigham and Women’s Hospital and Harvard Medical School; Columbia University Irving Medical Center; Brigham and Women’s Hospital and Harvard Medical School; Brigham and Women’s Hospital and Harvard Medical School

**Keywords:** alpha-synuclein, Parkinson’s disease, cellular assay, synucleinopathy

## Abstract

Parkinson’s disease (PD) is a progressive neurodegenerative disorder that affects over 12 million people worldwide. A central pathological feature is the accumulation of aggregated alpha-synuclein (αS) in Lewy bodies and Lewy neurites. Engineered αS variants such as 3K (E35K + E46K + E61K) and KLK (KTKEGV→KLKEGV in 6 repeats) have been shown to enhance membrane binding and aggregation propensity, contributing to cellular toxicity. To further investigate the impact of 3K and KLK on αS biology, we developed a sensitive assay in a human neuroblastoma model to assess expression levels and cytotoxicity. Relative to wild-type αS, the 3K mutant exhibited reduced steady-state expression and increased toxicity, consistent with prior reports. In contrast, the KLK mutant showed no marked change in protein expression but induced significantly higher toxicity, more than the 3K variant. These findings underscore the utility of our assay in dissecting disease-relevant mechanisms and highlight the potential of engineered αS variants to model pathogenic features of PD. This platform offers a versatile tool for evaluating therapeutic strategies targeting αS aggregation and toxicity in PD and related synucleinopathies.

## Introduction

Parkinson’s disease (PD) is a growing public health challenge as the second most common neurodegenerative disorder, currently affecting over 12 million people worldwide. Its prevalence has doubled over the past 25 years, with PD-related disability and mortality increasing faster than for any other neurological condition (GBD 2016 Neurology Collaborators, 2019). Projections indicate that by 2050, more than 25 million people will be living with PD, a 112% increase from 2021 (Su et al., 2025). While increasing age remains the primary risk factor, PD can also affect younger people (Bloem et al., 2021). Despite substantial development of therapeutic interventions for PD, none have been shown to slow disease progression. Consequently, the development of novel treatment strategies aimed at halting or delaying progression remains critical to alleviating the burden on patients, caregivers, and society.

A central pathological hallmark of PD and related synucleinopathies is the accumulation of abnormal intraneuronal aggregates containing alpha-synuclein (αS) (Spillantini et al., 1997). αS is a 14-kDa protein, encoded by the *SNCA* gene, and is predominantly expressed in the brain and enriched at presynaptic terminals, where it facilitates the release and reuptake of neurotransmitters, synaptic vesicle trafficking, and membrane curvature maintenance (reviewed by Bendor et al., 2013). It maintains a tightly regulated equilibrium between a cytosolic state and transient membrane association in neurons (Ramalingam & Dettmer, 2021; reviewed by Yeboah et al., 2019). Excessive αS-membrane interactions are implicated in αS aggregation (reviewed by Li & Dettmer, 2024). N-terminal point mutations in αS, such as E46K (Zarranz et al., 2004) and duplications or triplications (Chartier-Harlin et al., 2004; Singleton et al., 2003) of the *SNCA* locus have been associated with autosomal dominant forms of familial PD. All mutations are dominant, suggesting that there is a gain of function and that there may be a link between increased αS levels and PD pathogenesis. Increased αS levels and resulting aggregation can induce cytotoxicity through the formation of cytoplasmic inclusions, disruption of membrane integrity, and impairment of cellular processes such as vesicle trafficking and neurotransmission. Downstream events may include mitochondrial oxidative stress, impaired protein degradation pathways, and neuroinflammation (reviewed by Calabresi et al., 2023).

Robust and sensitive assays for quantifying αS levels and αS-associated toxicity are essential for understanding the role of αS in disease progression and to evaluate the efficacy of disease-modifying therapeutic interventions. We developed a bicistronic strategy that enables simultaneous monitoring of αS levels and cellular toxicity within live cells. Bicistronic constructs incorporating internal ribosome entry site (IRES) elements allow cap-independent translation of a second open reading frame from a single mRNA transcript. Since their discovery in viral RNAs, IRES elements have been widely utilized in experimental and pharmaceutical applications to co-express multiple proteins in live cells (Jang et al., 1988; Pelletier & Sonenberg, 1988). This strategy also allows the visualization of cells expressing the protein of interest without modifying the protein itself with a fluorescent tag (San Gil et al., 2017). Our αS-IRES-mCherry approach enables the simultaneous monitoring of αS expression and cytotoxicity, providing a more comprehensive view of αS-associated cellular dynamics (Li et al., 2025). The translation of a single mRNA molecule results in the expression of two proteins, the αS variant and the fluorescent reporter mCherry. mCherry is a red fluorescent protein widely used for live cell imaging due to its monomeric structure, high brightness, fast maturation, and good photostability, enabling visualization of protein dynamics and localization in live cells (Shaner et al., 2004). Our αS-IRES-mCherry assay represents a simple adaptation designed for dynamic, quantitative analysis of αS expression and cytotoxicity in parallel. Assessing αS levels normalized to mCherry by Western blot enable an accurate assessment of relative protein stability/half-life that accounts for any toxicity of the expressed variant. Monitoring the time-course of mCherry fluorescence in the culture by live-cell microscopy, on the other hand, is a reliable readout for the toxicity associated with the respective variant.

Using this approach, we compared the protein levels and toxicity of WT αS with the engineered 3K and KLK variants. The αS 3K variant (E35K + E46K + E61K) amplifies the E46K mutation linked to familial PD, whereby the additional positively charged lysine residues may increase the interaction with the negatively charged lipid head groups (reviewed by Dettmer, 2018). Another engineered mutant of αS is KLK, containing six substitutions (KTKEGV→KLKEGV) in the N-terminal repeats of the protein. This increases the hydrophobicity of the hydrophobic half of αS’s amphipathic helix, thereby stabilizing its membrane association (Dettmer et al., 2017). Compared to WT αS, the 3K mutant exhibited reduced steady-state expression based on Western blot analyses. Nonetheless, increased toxicity was observed based on reduced mCherry signal in the culture. 3K may be more toxic per molecule as it amplifies the familial PD-linked E46K mutation, which has been shown to cause increased cytotoxicity without altering protein aggregation (Íñigo-Marco et al., 2017). The KLK mutant displayed WT-like expression levels and enhanced toxicity even higher than the 3K mutant. The KLK mutant showed even more reduced steady-state expression and increased toxicity compared to the 3K mutant. Our findings align with prior reports, emphasizing the potential of these engineered variants to model the pathological characteristics of αS in PD. This platform, therefore, offers a valuable tool for evaluating therapeutic strategies aimed at reducing αS aggregation and/or toxicity in PD and related synucleinopathies.

## Materials and Methods

All materials mentioned were purchased from Invitrogen unless stated otherwise.

### Cell Culture

BE(2)-M17 human neuroblastoma cells (M17D) were maintained in Dulbecco’s Modified Eagle Medium (DMEM) supplemented with 10% fetal bovine serum (Sigma) and 2 mM L-glutamine, under standard culture conditions of 5% CO_2_ at 37°C. For transfection experiments, cells were seeded into 48- or 96-well plates and transfected using Lipofectamine 2000, following the manufacturer’s protocol. Post-transfection, cells were collected at 24 h, 48 h, and 72 h for downstream analysis.

#### cDNA Cloning

**Figure 1:**
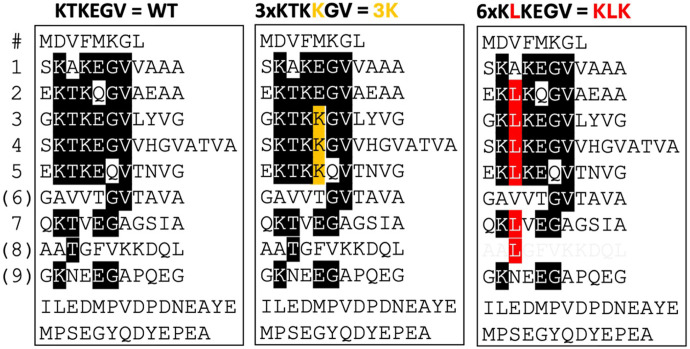
Schematic of WT, 3K, and KLK human αS amino acid sequences, aligned by the KTKEGV motifs. Amino acids that fully conform to “KTKEGV” are highlighted in black. Repeats 1–9 are interrupted only once by “ATVA” between repeats 4 and 5. In the 3K variant, lysine substitutions are highlighted in orange, whereas in the KLK variant, leucine substitutions are highlighted in red

αS variants were cloned into the pLVX-EF1α-IRES-mCherry plasmid (Clontech) by restriction-enzyme-based cloning using SpeI/NotI sites (Fig. 1), resulting in αS-3K or KLK-IRES-mCherry plasmid. αS-WT-IRES-mCherry plasmid was described previously (Dettmer et al., 2017). The amino acid sequences of the αS variants are shown in Fig. 1.

### Cell Lysis

M17D cells were centrifuged at 3,000 *g* for 2 min at room temperature, and the culture media was aspirated. The pellet was resuspended in PBS, centrifuged again at 3,000 *g* (room temperature; 2 min), and the PBS was aspirated. Cells were lysed on ice using 75 μL of 0.5% Triton X-100 with protease and phosphatase inhibitors. After incubation, samples were centrifuged at 16,100 *g* for 15 min at 4 °C. 15 μL of 4x protein sample loading buffer (Li-Cor) was added to new 1.5 mL tubes. 45 μL of the supernatant was added, boiled for 3 min, and centrifuged at 3,000 *g* for 2 min.

### Immunoblotting

12 μL of lysate were loaded per lane. Samples were electrophoresed on NuPAGE 4–12% Bis-Tris gels with NuPAGE MES-SDS running buffer and SeeBlue Plus2 marker. Gels were electroblotted onto nitrocellulose membranes using an iBlot 2NC stack and iBlot 2 device (20 V for 1 min, 23 V for 4 min, then 25 V for 2 min). Membranes were incubated in 0.4% paraformaldehyde for 20 min, rinsed with TBS, stained with 0.1% Ponceau S in 5% acetic acid, rinsed with water and blocked in the blocking buffer (Li-Cor blocking buffer ‘TBS’) for 30 min at room temperature. After blocking, membranes were incubated in primary antibody in blocking buffer for either 1 h at room temperature or overnight at 4 °C. Membranes were washed 3 × 10 min in TBS-T or PBS-T at room temperature and incubated in secondary antibody in blocking buffer for 40 min at room temperature. Membranes were then washed 3 × 10 min in TBS-T or PBS-T and scanned (Odyssey CLx, Li-Cor).

### Antibodies

Primary antibodies in Western blotting were monoclonals 15G7 to αS (Enzo Life Sciences; 1:250 in Western blot), AB9484 to GAPDH (Abcam; 1:3,000 in Western blot), and 16D7 to mCherry (Thermo Scientific; 1:300 in Western blot). Secondary antibodies were polyclonal Li-Cor IRDye anti-mouse, antirabbit, and anti-rat (926-68070, 926-68071, and 926-32219 respectively; 1:10,000 in Western blot).

### Statistical Analysis

We performed unpaired two-tailed t-tests to compare WT and engineered 3K and KLK αS mutants using GraphPad Prism version 10 following the program’s guidelines. Normal (Gaussian) distribution was confirmed for all values in bar graphs by Shapiro-Wilk and Kolmogorov-Smirnov tests. Graphs represent means ± standard deviation (SD).

### Live-cell Imaging

Cells were incubated in the Incucyte S3-C2 system (Sartorius). Images from phase-contrast and red fluorescence channels were taken every 4 h using a 10× objective. Red fluorescence integrated intensity and phase-channel cell confluence in % were quantified using the program’s standard internal processing definitions. Data from different wells of a 48- or 96-well plate (biological replicates) were averaged, and 3 independent experiments were performed on 3 different days. Graphs represent mean ± standard deviation (SD). Statistical analyses were performed using GraphPad Prism as described above.

## Results

### Establishing a Robust Assay in Neuroblastoma Cells

We aimed to develop a robust cellular assay with two distinct readouts: (1) detection and relative quantification of protein levels of αS variants, and (2) assessment of their relative toxicity.

**Figure 2:**
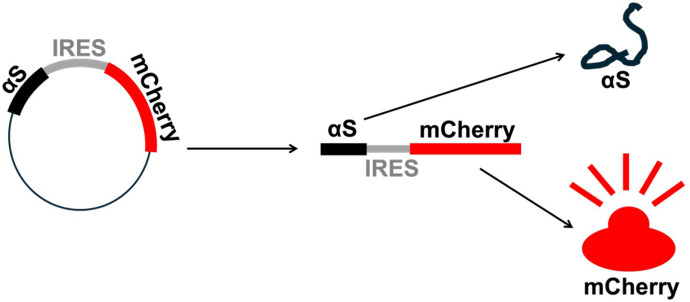
Schematic of cDNA plasmid. The plasmid encodes αS and mCherry, separated by IRES, as a bicistronic construct. One plasmid results in a single mRNA transcript that produces both αS and the red fluorescent reporter protein mCherry

We used cDNA plasmids that encode αS and mCherry as a bicistronic construct, with the genes separated by an internal ribosomal entry site (IRES). This design allows both genes to be transcribed from the same promoter into a single mRNA, resulting in αS and mCherry co-expression in the cells. The expression of mCherry serves as an intrinsic control for transfection efficiency, with Western blotting used to quantify its levels. Additionally, mCherry fluorescence in live cells was monitored as a proxy for αS-associated toxicity using live-cell fluorescence microscopy (Incucyte). A toxic αS variant would lead to reduced cell proliferation and increased cell death, thereby decreasing red mCherry fluorescence, which can be tracked in real time to assess toxicity (Fig. 2).

We first identified a set of conditions that produced reliable and interpretable results. For Western blot analysis of αS levels, we plated cells in 48- and 98-well plates such that they would reach 50–80% confluence on the day of transfection. (Lipofectamine 2000). For robust immunodetection, we identified monoclonal antibodies against αS (rat 15G7), GAPDH (mouse EPR6256), and mCherry (rabbit 16D7). This antibody combination provided high sensitivity and signal specificity, enabling reliable quantification of protein abundance and toxicity without artifacts, independent of transfection efficiency (see Materials and Methods section for details).

To assess cell viability over time, M71D cells were transfected at approximately 50% confluence in 48- and 96-well plates and monitored longitudinally for five days using live-cell imaging. This was followed by Incucyte quantitative analysis of mCherry fluorescence and bright field signals.

### Incucyte Live-Cell Microscopy Validates Transfection via mCherry Expression

**Figure 3:**
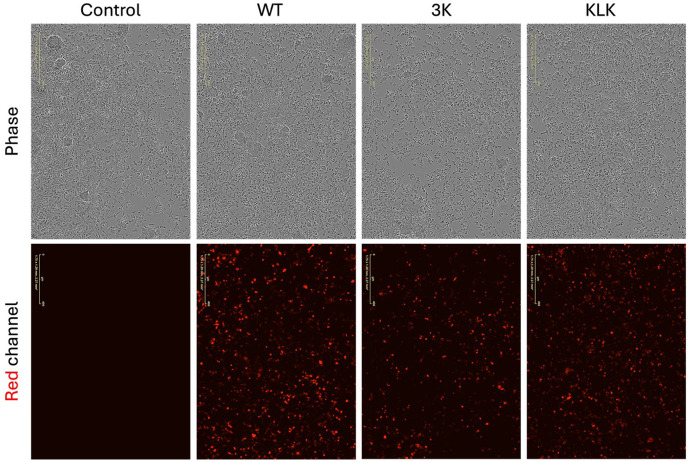
Validation of transfection efficiency using Incucyte. Equally scaled, representative epifluorescence images images of M17D neuroblastoma cells transfected with plasmids encoding αS, mock (−) or WT, and the red fluorescent mCherry reporter (control: mock transfection without plasmid). Phase channel shows M17D cells without fluorescence. Red channel displays mCherry signal, serving as a proxy for transfection and cell viability. Scale bar, 200 μm

Before evaluating the effect of mutations on protein levels and cytotoxicity, we first validated the correlated expression of αS and the red fluorescent reporter protein mCherry. We visualized the red mCherry fluorescence by live-cell imaging using the Incucyte system 24 h after transfection. As expected, control M17D neuroblastoma cells exhibited no visible red fluorescence, whereas cells transfected with the respective αS constructs displayed robust mCherry expression (Fig. 3). An estimated > 90% of the cells showed detectable mCherry signal.

### Levels of WT compared to 3K and KLK αS

**Figure 4:**
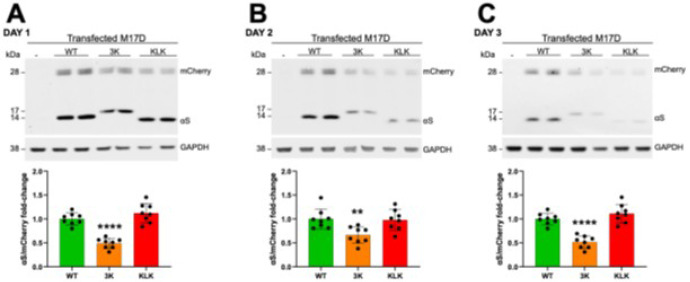
Expression levels of WT, 3K, and KLK αS over time. Western blot analysis of mCherry, total αS, and GAPDH in M17D cells transfected with WT, 3K, and KLK variants. (**A-C**) Quantification of total αS relative to WT, normalized to mCherry at 24 h (**A**), 48 h (**B**), and 72 h (**C**) post-transfection. Data represent *N* = 4 independent experiments with *n* = 8 biological replicates per condition. Graphs show mean ± S.D. Normal distribution was confirmed for all values in bar graphs using Shapiro-Wilk and Kolmogorov-Smirnov tests. Unpaired t-test, two-tailed. Criteria for significance: *p < 0.05, **p < 0.01, ***p < 0.001, ****p < 0.0001

We investigated the protein expression of WT, 3K, and KLK αS by transfecting M17D cells with mock (-), WT αS, 3K αS, and KLK αS constructs. Western blotting was performed to quantify total αS, mCherry and GAPDH (loading control) levels (Fig. 4). KLK αS expression was comparable to WT, whereas 3K αS exhibited reduced protein levels relative to mCherry, with significant decreases on day 2 (*p* < 0.01) and on days 1 and 3 (*p* < 0.0001) after transfection.

In addition, we observed that the mCherry signal appeared as a doublet for all αS variants (Fig. 4). This may be explained by the presence of an alternative translation initiation site in the mCherry coding sequence (Fages-Lartaud et al., 2022). Translation from this site generates a slightly truncated protein isoform (around 1–2 kDa smaller). Both isoforms are stable, fluorescent, and antibody-reactive, and both signals were included in our analyses.

### Toxicity of WT compared to 3K and KLK αS

**Figure 5:**
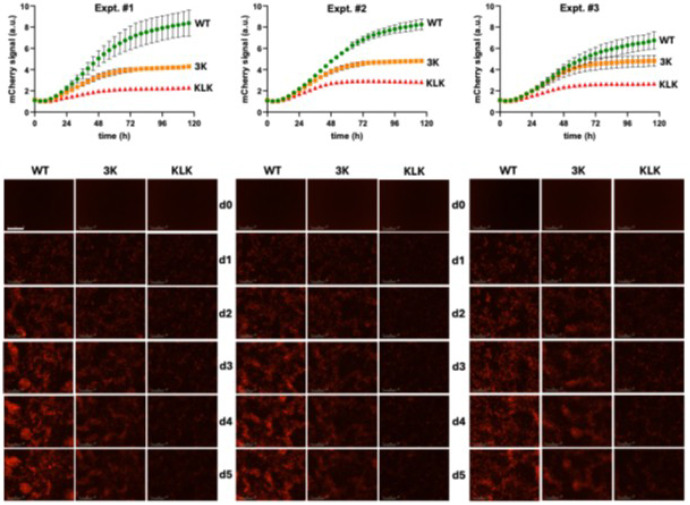
Toxicity of WT, 3K, and KLK αS. Time-course quantification of mCherry fluorescence signal (arbitrary units, a.u.), normalized to control, measured with Incucyte in M17D cells expressing WT, 3K, or KLK αS. Time points indicate hours post-transfection. Graphs represent *N* = 3 independent experiments over 5 days. Each time point represents *n* = 5 biological replicates. d0 = day 0 (pre-transfection), d1 = day 1 after transfection, etc. Mean ± SD. Scale bar, 200 μm

Next, we investigated the toxicity of WT αS and the engineered mutants 3K and KLK. M17D cells were transfected with WT, 3K, and KLK αS, and we used live-cell fluorescence microscopy with the Incucyte system to measure the mCherry fluorescence signal over a period of 5 days. As αS and mCherry were co-expressed bicistronically in the cDNA plasmids, mCherry fluorescence served as a real-time proxy of αS-associated toxicity. In three independent experiments, the mCherry signals of 3K and KLK became significantly lower than WT αS over time (Fig. 5). This is supported by previous work suggesting that 3K and KLK are more toxic than WT αS, with KLK being the most toxic to cells (Dettmer et al., 2015b).

## Discussion

This study presents a dual-readout assay designed to quantify αS expression and evaluate its associated toxicity in a human neuroblastoma model. The approach was employed to compare human αS WT with 3K and KLK variants. A bicistronic αS-IRES-mCherry construct is integrated with live-cell imaging, providing simultaneous monitoring of both protein levels and cell viability over time. By using mCherry as an internal control for transfection efficiency and cell health, this approach offers a robust normalization strategy, minimizing variability and improving data interpretation. Bicistronic expression systems using IRES elements are well-established in research (Jang et al., 1988; Pelletier & Sonenberg, 1988). For instance, they have been used to co-express untagged proteins with fluorescent reporters (San Gil et al., 2017). However, to our knowledge, this is the first application of an IRES-based bicistronic system pairing αS with mCherry for time-resolved quantification of both protein expression and toxicity. Compared to conventional approaches, such as endpoint viability assays or Western blotting lacking an intrinsic protein translation control, our strategy offers several advantages. Live-cell imaging enables temporal resolution that is unavailable in static assays, while co-expression of the mCherry reporter provides an intrinsic normalization control across experimental conditions and replicates. These features contribute to enhanced sensitivity and reproducibility. Certain caveats of IRES-based bicistronic systems must be considered, although these are unlikely to have affected our findings. IRES-mediated translation can vary between cistrons, with the expression level of the downstream cistron usually lower and more variable than the upstream cistron, and can depend on cell type, species, and physiological state (Hennecke et al., 2001; Mizuguchi et al., 2000). Different IRES types can have varying levels of flexibility regarding the insertion site of a gene of interest, which can limit the engineering of some IRES-based systems (Borman et al., 1995). The level of gene expression in lentiviral vectors can also depend on the gene’s position within the construct and the total number of genes expressed (Chinnasamy et al., 2006). Alternative strategies, such as 2A peptide sequences (Kim et al., 2011; Szymczak et al., 2004), can provide more stoichiometric co-expression, but may also introduce extraneous amino acids (“scar”) into the protein of interest. Additionally, the half-life of mCherry can be very long (Bell et al., 2025), which could obscure certain effects when monitoring toxicity.

In our assay, the 3K αS mutant exhibited reduced steady-state expression accompanied by increased toxicity relative to WT αS (Figs. 4 and 5). In contrast, the KLK variant showed no significant alteration in steady-state expression yet induced markedly greater toxicity compared to WT, exceeding that observed for the 3K mutant (Figs. 4 and 5). This aligns with prior studies showing that both 3K and KLK mutants may be toxic *in vivo*. These engineered variants shift the normally highly soluble, cytosolic αS toward insoluble, membrane-associated species. This causes αS to accumulate at membranes, inducing acute cytotoxicity, and the formation of round cytoplasmic inclusions linked to neurotoxicity (Dettmer et al., 2015a; Dettmer et al., 2015b). While both 3K and KLK mutations enhance αS-membrane interactions, KLK exerts a markedly stronger effect on toxicity by increasing the hydrophobicity and stability of the N-terminal amphipathic helix. This stabilization promotes tighter membrane association and the formation of dense, vesicle- and lipid-rich inclusions, resulting in substantially greater cytotoxicity, despite steady-state αS expression levels remaining comparable to WT. These findings suggest that KLK may drive toxicity primarily by altering αS-membrane interactions and aggregation behavior rather than by elevating total protein expression. Such membrane-mediated effects are likely to facilitate conformational changes that accelerate oligomerization and membrane-rich aggregation, consistent with prior work indicating that PD pathogenesis is more strongly linked to aggregation kinetics and toxic oligomer formation than to total αS levels (Flagmeier et al., 2016; Tosatto et al., 2015). Taken together, the increased toxicity observed for 3K and KLK highlights the sensitivity of the assay to detect pathogenic differences in αS-membrane association and toxicity over time.

While this assay is a valuable screening platform, it does not capture the complexity of mature neurons and pathogenesis in an intact organism, which would need systems where αS is expressed in the brain. Additionally, transient cDNA transfection creates a non-steady-state condition. This leads to the immediate production of high levels of encoded proteins followed by the progressive decline of transgene transcription as the DNA becomes diluted and degraded in dividing cells. The observed effects may therefore differ in viral overexpression systems, primary cultures, iPSC-derived neurons, or *in vivo* paradigms. Future applications of this assay could extend to the analysis of additional familial PD mutations, such as A53E and G51D (Lesage et al., 2013; Pasanen et al., 2014), *SNCA* multiplications, and pharmacological modulators targeting αS expression and clearance. Integrating with iPSC-derived neuronal models or *in vivo* models would also provide further mechanistic insights.

## Conclusions

In summary, we establish the αS-IRES-mCherry system as a robust and versatile platform for quantitative, dynamic analysis of αS expression and toxicity in living cells. The engineered 3K and KLK mutants were designed to enhance membrane association rather than to intrinsically promote aggregation (Dettmer et al., 2017; Volles & Lansbury, 2007). By distinguishing αS variants with pathogenic relevance, this assay facilitates mechanistic dissection of αS-membrane interactions and provides a framework for the screening and optimization of therapeutic strategies targeting αS-mediated aggregation and neurotoxicity in PD and related synucleinopathies.

## Data Availability

All data supporting the findings of this study are available within the paper and its Supplementary Information
